# Characterization of the Kenaf (*Hibiscus cannabinus*) Global Transcriptome Using Illumina Paired-End Sequencing and Development of EST-SSR Markers

**DOI:** 10.1371/journal.pone.0150548

**Published:** 2016-03-09

**Authors:** Hui Li, Defang Li, Anguo Chen, Huijuan Tang, Jianjun Li, Siqi Huang

**Affiliations:** Institute of Bast Fiber Crops, Chinese Academy of Agricultural Sciences, Changsha, Hunan, China; Georgia Institute of Technology, UNITED STATES

## Abstract

Kenaf (*Hibiscus cannabinus* L.) is an economically important natural fiber crop grown worldwide. However, only 20 expressed tag sequences (ESTs) for kenaf are available in public databases. The aim of this study was to develop large-scale simple sequence repeat (SSR) markers to lay a solid foundation for the construction of genetic linkage maps and marker-assisted breeding in kenaf. We used Illumina paired-end sequencing technology to generate new EST-simple sequences and MISA software to mine SSR markers. We identified 71,318 unigenes with an average length of 1143 nt and annotated these unigenes using four different protein databases. Overall, 9324 complementary pairs were designated as EST-SSR markers, and their quality was validated using 100 randomly selected SSR markers. In total, 72 primer pairs reproducibly amplified target amplicons, and 61 of these primer pairs detected significant polymorphism among 28 kenaf accessions. Thus, in this study, we have developed large-scale SSR markers for kenaf, and this new resource will facilitate construction of genetic linkage maps, investigation of fiber growth and development in kenaf, and also be of value to novel gene discovery and functional genomic studies.

## Introduction

Kenaf (*Hibiscus cannabinus* L.) is an important crop plant species that is grown for its fiber, which is present in the stem bast. The fiber is smooth, has antibacterial properties and has excellent air permeability. Kenaf has multiple uses, such as in paper pulp, carpet backing, building materials, and forage for livestock [1–4]. It is currently cultivated in more than 20 countries, particularly in China, India, and Thailand [5].

As the plant has high economic potential, considerable efforts are being made to improve the fiber quality and fiber yield of kenaf. However, improvements in fiber traits and yield have been hindered by the lack of genomic information such as detailed genetic maps, and of quantitative trait loci (QTLs) for fiber-related traits.

In recent years, random amplified polymorphic DNA (RAPD)[6], inter simple sequence repeat (ISSR) [7,8], and sequence-related amplified polymorphism (SRAP) markers[9,10] have been developed. These markers have been used to analyze genetic diversity and expand the genetic linkage map in kenaf.

Simple sequence repeat(SSR) markers are among the most valuable of genomic sequences that can be used in plant genetic analyses. SSR markers are abundant and unevenly distributed throughout the genomes of eukaryotes. ISSR markers are amplified using a single primer based on an SSR motif and anchored by a 2 to 4 degenerate nucleotide sequence at the 5′ or 3′ ends. The primers amplify 100 to 3000bp sequences between inversely oriented closely spaced microsatellites. The PCR products are therefore anonymous SSR loci. ISSR markers have dominant inheritance and do not have locus specificity. They are especially suitable for phylogenetic studies, genetic diversity analyses, and cultivar identification. By contrast, SSRs have locus specificity, reliability, co-dominant inheritance, and significant polymorphism and have been widely used in genetic mapping and molecular breeding studies in plants.

SSRs can be divided into genomic SSRs (derived from random genomic sequences) and EST-SSRs (derived from ESTs) depending on their origin. EST-SSRs are derived from coding sequences and are therefore tightly linked to functional genes that may influence important agronomic characters. Thus, EST-SSRs are among the most important genetic markers for the analysis of genetic diversity, high-density genetic mapping, and marker-assisted breeding [11].

Traditional methods to isolate and identify EST-SSRs are labor intensive, time consuming, and costly [12,13]. However, advances in sequencing technologies, such as next-generation sequencing, have facilitated the identification of SSR loci derived from ESTs and made it feasible to identify EST-SSRs in any species[14–16].

The aim of the present study was to generate a large-scale EST database, develop a set of EST-SSRs, and evaluate the quality of these novel SSR markers. The EST database was generated by transcriptome sequencing of the kenaf genome using an Illumina paired-end sequencing platform. To our knowledge, this is the first study to characterize EST-SSRs and develop SSR markers in kenaf. The data reported here will provide a valuable resource for genetic diversity analysis, construction of genetic linkage maps, and for marker-assistant breeding in kenaf.

## Materials and Methods

### Sample collection and preparation

The kenaf cultivar “Zhong hong ma 16” was grown in the experimental field of the Institute of Bast Fiber Corps, Chinese Academy of Agricultural Sciences Changsha, China in 2014. Samples of tissues including leaf, root, stem shoot, stem tip and stem bast were collected, frozen immediately in liquid nitrogen, and stored at -70°C until use.

### RNA extraction and cDNA library preparation and sequencing

Total RNA was extracted from each tissue sample using TRIzol reagent according to the manufacturer’s protocol (Invitrogen, Camarillo, CA). The quality of the total RNA was verified using an Agilent 2100 Bioanalyzer before further processing. Equal volumes of RNA from each of the five tissues were mixed for sequencing; the sequencing was performed at the Beijing Genomics Institute (BGI; Shenzhen, China). Poly(A) mRNA was isolated from total RNA with Magnetic Oligo(dT) Beads. First, the purified mRNA was fragmented into small pieces, and then double-stranded cDNA was synthesized using the SuperScript Double-Stranded cDNA Synthesis kit (Invitrogen) with the random hexamer (N6) primer (Illumina). The double-stranded cDNA was subjected to end-repair and phosphorylation using T4 DNA polymerase, the Klenow DNA polymerase, and T4 polynucleotide kinase. These repaired cDNA fragments were 3′-adenylated using Klenow 3′ to 5′ exo-polymerase, then ligated with Illumina paired-end adapters to the end of these fragments using T4 DNA ligase. The adapter-ligated fragments were separated on an agarose gel and cDNA fragments of approximately 200 ±25 bp were excised from the gel. In order to enrich the purified cDNA template, PCR was performed to amplify the cDNA fragments. The cDNA library was constructed with a fragment length of 200 ±25 bp. After validation with an Agilent 2100 Bioanalyzer, the cDNA library was sequenced on a PE flow cell using an Illumina Hiseq2000 sequencing platform. Three duplicate cDNA libraries were constructed and sequenced separately using an Illumina Hiseq2000 genome analyzer to minimize systematic biases and random error in sequencing and allow for the detection of low-abundance transcripts.

### Data filtering and assembly

Before assembly of the kenaf transcriptome, a stringent filtering process was carried out. The adapter sequences of the raw reads were removed, and low-quality sequences (reads with ambiguous bases ‘N’) and reads with more than 20% Q<20 bases were also removed. The sequencing data was deposited in NCBI Sequence Read Archive database under accession number is SRP 063749. Assembly of the clean reads was performed using Trinity software. Trinity combines reads that have overlapping nucleic acid sequences to form contigs. Then, contigs from the same transcript are assembled and sequences that cannot be extended at either end are defined as unigenes. TGICL software was used to eliminate redundant unigenes and for further assembly of unigenes to form a single non-redundant set. Unigenes from all three libraries were assembled again to acquire non-redundant unigenes (All-Unigene set) that were as long as possible. The Assembly project has been deposited at DDBJ/EMBL/GenBank under accession number GEED00000000.

### Gene annotation

The All-Unigene set was aligned to the NCBI non-redundant (NR) protein database and the Swiss-Prot protein database using blastx with an E-value cut off of 10^−5^. Unigenes that could not be aligned were scanned by ESTScan [17]. Based on NR annotation we used the Blast2GO program[18] to obtain GO annotation of unigenes and WEGO software[19] for GO functional classification of all unigenes; these analyses provided information on the distribution of kenaf gene functions. Unigene sequences were also aligned to the COG database to predict possible function, and to determine the gene function distribution characteristics of kenaf. The KEGG database was used to obtain pathway assignments [20].

### EST-SSR detection, primer design, and selection

Mining for potential SSRs among the 71,318 unigenes was performed using MISA software [21]. The default criteria were adjusted to identify a minimum of 6 repeats for dinucleotide motifs, 5 repeats for trinucleotide motifs, 4 repeats for tetranucleotide motifs, 4 repeats for pentanucleotide motifs, and 4 repeats for hexanucleotide motifs. Primer pairs were designed using Primer3[22]. The major parameters for primer pair design were set as follows: primer length, 18–23 bp, PCR product size 100–400 bp, GC content 40–70%, and annealing temperature 50–60°C. Primers designed using these criteria were further filtered as follows: no SSRs in the primer; alignment of the primers to the unigene sequence allowed 3 mismatches at the 5′ site and 1 mismatch at the 3′ site; primers that aligned to more than one unigene were discarded. The SSRs on the product sequences were identified using ssr_finder. Products that gave the same result with ssr_finder as MISA were kept.

### Survey of EST-SSR polymorphism

Twenty-eight kenaf varieties including Chinese landraces, cultivars, wild species and near marginal species, were selected for investigation of their polymorphisms using the EST-SSRs. Genomic DNA was isolated using the CTAB method. PCR amplification was performed using a final volume of 10 μl containing 10 ng template DNA, 2× PCR Master Mix, and 4 μM of each primer. The following amplification protocol was applied: incubation at 94°C for 5 min, then 33 cycles of 94°C for 39 s, 55°C for 45 s, 72°C for 1 min, and a final extension at 72°C for 10 min, before a final 4°C hold. The PCR products were separated on an 8% polyacrylamide gel with a 50 bp DNA marker to calculate the length of the EST-SSR amplicons. Gels were stained with 0.1% silver nitrate as previously described[23], and photographed in white light.

## Results

### Illumina paired-end sequencing and assembly

To obtain a global overview of the kenaf transcriptome and generate a broad survey of genes associated with kenaf vegetative growth and development, total RNAs were extracted from the vegetative tissues (leaf, root, stem shoot, stem tip, and stem bast), and an equal volume of each was pooled. To minimize bias from the Illumina sequencing and transcriptome sampling, we constructed 3 cDNA libraries from the pooled RNA sample and sequenced these separately using an Illumina Hiseq2000 genome analyzer.

Each sequenced sample yielded 2 × 90 bp independent reads from either end of a cDNA fragment, using Illumina Hiseq2000 sequencing platform. The three 200 bp insert libraries gave 52.9, 51.9, and 51.9 million raw sequencing reads. After stringent quality assessment and data cleaning, 48.3, 47.7, and 47.6 million high quality reads were obtained with 97.77% Q20 bases (Table 1).

**Table 1:** Output statistics of sequencing.

Sample	Total raw reads	Total clean reads	Total clean nucleotides	Q20 (%)	N (%)	GC (%)
1	52,922,062	48,326,998	4,349,429,820	97.70	0.00	46.05
2	51,859,144	47,656,960	4,289,126,400	97.77	0.00	45.42
3	51,881,472	47,594,544	4,283,508,960	97.7	0.00	45.51

The high quality reads were assembled into 113,478 contigs with an average length of 358 nt, 116,009 contigs with an average length of 369 nt, and 119,936 contigs with an average length of 368 nt, respectively. The length distribution of the contigs in each library is shown in S1 Fig. Length distribution of contigs The N50 was 632 nt, 688 nt, and 681 nt for each library, respectively.

Using a paired-end sequencing strategy, the paired-end reads were aligned to contigs and then contigs from the same transcript were assembled; if the sequence was not extended at either end, the contigs were defined as unigenes. The assembly from the three libraries yielded 63,042 unigenes with an average length of 670 nt, 64,502 with an average length of 771 nt, and 66,895 with an average length of 760 nt. The length distributions of the unigenes in each library are shown in S2 Fig. Length distribution of unigenes The N50 of the three libraries was 1217 nt, 1465 nt, and 1448 nt, respectively. Unigenes from the three libraries were pooled and assembled into non-redundant unigenes for further analysis. Overall, the contig and unigene length distributions in the three libraries were consistent, indicating that the Illumina sequencing was reproducible and reliable.

In total, 71,318 unigenes with an average length of 1143 nt, an N50 of 1784 nt, and a total length of 81,509,256 nt were obtained. This total included 13,818 unigenes (19.38%) of less than 300 nt, 54,085 (75.84%) with lengths from 301 nt to 3000 nt, and 3415 (4.78%) with lengths greater than 3000 nt S3 Fig. Length distribution of all unigenes

### Functional annotation by searching against public databases

For analysis of function, the assembled unigenes were annotated using the National Center for Biotechnology Information (NCBI) non-redundant protein (Nr) database, the COG database, the Swiss-Prot database, and the KEGG database using the blastx algorithm and specifying an E-value threshold of 10^−5^. Additionally, a sequence similarity search was performed using the NCBI Nt (non-redundant nucleotide) database using blastn with an E-value threshold of 10^−5^. We found that of the 71,318 unigenes, 56,147 (78.72%), 38,065 (53.37%), 33,807 (47.40%), 22,049 (30.92%), and 51,851 (72.7%) showed significant similarity to known proteins in the Nr, Swiss-Prot, KEGG, COG and Nt databases, respectively (Table 2).

**Table 2:** Results of annotation of unigenes to protein databases.

Sequence file	NR	NT	Swiss-Prot	KEGG	COG	GO	ALL
All-unigene.fa	56,147	51,851	38,065	33,807	22,049	45,855	58,095

**Fig 1:**
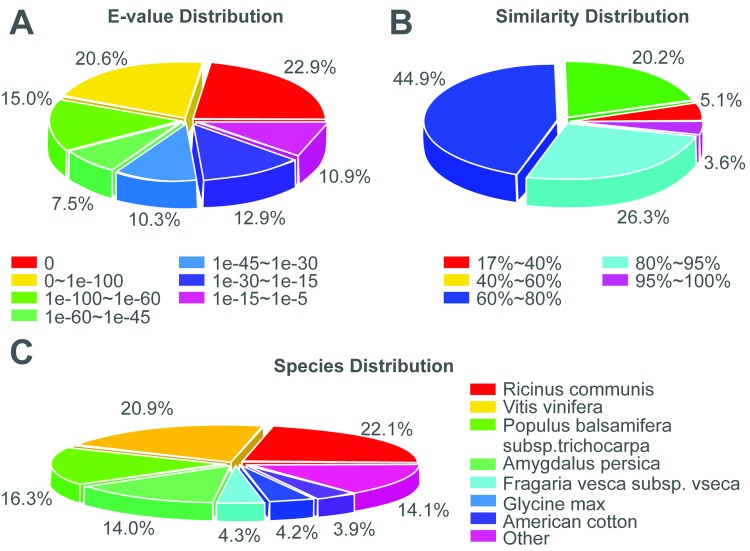
Characteristics of similarity of All-unigene against NR database. (A) E-value distribution of All-unigene. (B) Similarity distribution of All-unigene. (C) Species distribution of All-unigene.

Overall, 81.46% (58,095) unigenes were successfully annotated using the protein databases. The E-value distribution of the top hits in the Nr database revealed that 66.0% of the mapped sequences showed significant homology (less than 1.0E-4.5), and 74.8% and 29.9% of the sequences with similarities greater than 60% and 80% were found. Interestingly, 22.1% of the unigenes showed significant homology with sequences from *Ricinus communis*, and 20.9, 16.3, 14.0, 4.3, 4.2, and 3.9% showed homologies with *Vitis vinifera*, *Populus balsamifera* subsp. *trichocarpa*, *Amygdalus persica*, *Fragaria vesca* subsp. *vesca*, *Glycine max*, and American cotton sequences, respectively; 14.1% were unknown Fig 1.

Characteristics of similarity of All-unigene against NR database.(A) E-value distribution of All-unigene. (B) Similarity distribution of All-unigene. (C) Species distribution of All-unigene

### Functional classification by GO and COG

**Fig 2:**
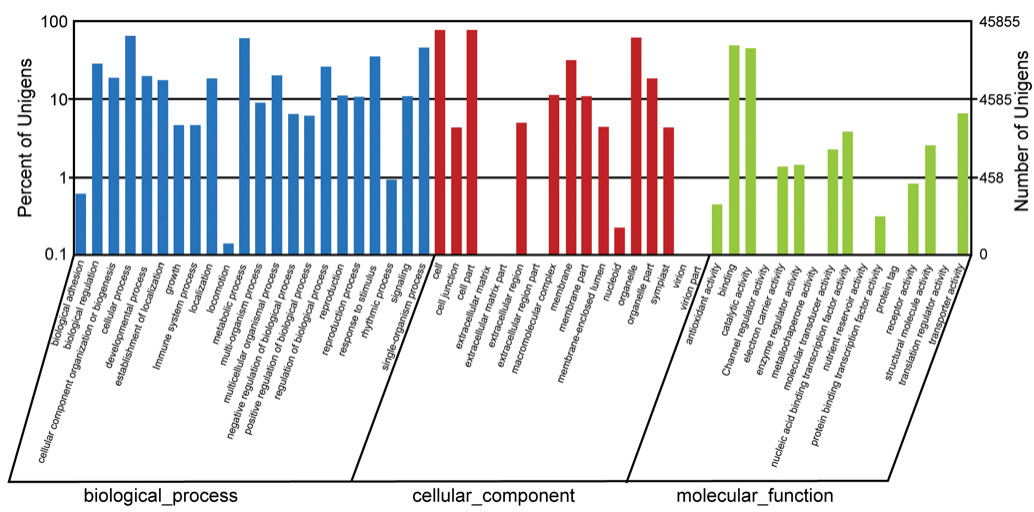
Gene Ontology (GO)classifications of All-unigenen. The results are summarized in three main categories: Biological process, Cellular component and Molecular function.

All unigenes were aligned to the COG and GO databases for functional prediction and classification. The Gene Ontology (GO) database has three principal categories: biological process, cellular component, and molecular function. On the basis of Nr annotation, 45,855 unigenes were assigned to these three categories, and were distributed across 45 functional categories. For biological process, 65.29% were cellular process unigenes, and 61.32% were metabolic process unigenes. For cellular component, 77.62% of unigenes were assigned to cell and 77.6% to cell part. For molecular function, 49.62% were binding unigenes and 45.32% were catalytic activity unigenes Fig 2. Gene Ontology (GO)classifications of All-unigenen. The results are summarized in three main categories: Biological process, Cellular component, Molecular function.

**Fig 3:**
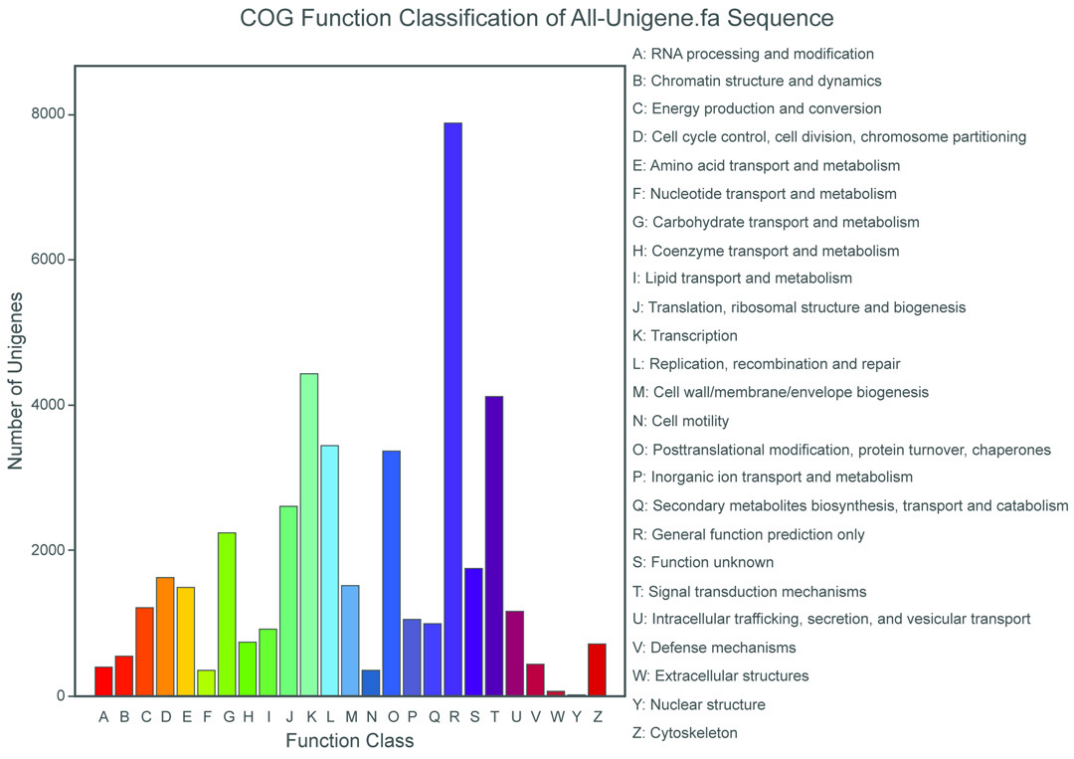
Clusters of orthologous groups(COG) classification of All-unigene.

The COG database is used to classify orthologous gene products. In total, 22,049 unigenes were assigned to the 25 COG categories. Of the 25 COG categories, the most frequently identified classes were general function (35.7%), transcription (20.1%), and signal transduction mechanisms (18.7%); a few unigenes were assigned to the nuclear structure and extracellular structures categories Fig 3. Clusters of orthologous groups(COG) classification of All-unigene.

### Metabolic pathway analysis by KEGG

The KEGG Pathway database records the networks of molecular interactions in cells and species-specific variations. Pathway-based analysis provides insight into the biological functions and interactions of genes. First, using blastx with an E-value threshold of 10^−5^, 33,807 unigene sequences were grouped in 128 KEGG pathways.

### Development of SSR markers

Next, we screened the 71,318 EST (unigene) sequences using MISA software to identify SSR loci. We found 12,886 SSRs in 10,892 sequences; 1673 of the EST sequences contained more than one SSR (Table 3).

**Table 3:** Results of microsatellite search.

Total number of sequences examined	71318
Total length of examined sequences (bp)	81509256
Total number of identified SSRs	12886
Number of SSR-containing sequences	10892
Number of sequences containing more than 1 SSR	1673
Number of SSRs present in compound formation	545

After filtering, 9324 primer pairs were successfully designed using Primer3 (S1 Table). The SSRs included 5752 trinucleotide motifs (61.7%), 2137 dinucleotide motifs (22.9%), 733 hexanucleotide motifs(7.9%), 405 pentanucleotide motifs(4.3%), 267 tetranucleotide motifs (2.9%) and 30 mononucleotide motifs(0.3%). The numbers of tandem repeats were calculated and are shown in S4 Fig.

### Analysis of polymorphisms in the SSR markers

To assess the quality of the SSR markers, 100 primers were randomly selected (S2 Table) and used to evaluate application and polymorphism in the 28 kenaf varieties (S3 Table). Stable and reproducible amplification products were produced by 72 of the primer pairs, and 60 of the 72 pairs identified polymorphisms. A total of 156 alleles were successfully amplified by the 72 SSR markers. The number of alleles per locus varied from 1 to 4. Among the 156 loci that were successfully amplified by the 72 SSR markers, 41 had two alleles, 17 had 3 alleles and 3 loci had 4 alleles in the 28 varieties; the remaining 11 loci showed no polymorphism among the 28 kenaf varieties.

### Evaluation of genetic relationships among 28 kenaf varieties

**Fig 4:**
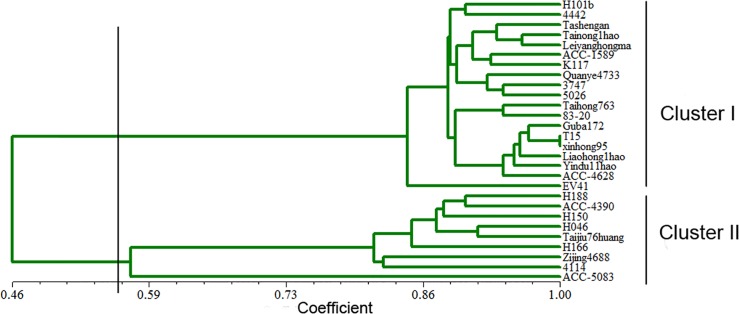
Dendrogram of 28 Kenaf varieties based on cluster analysis of 72 ploymorphic EST-SSR markers.

Based on the above results, 72 SSR primer pairs were used to evaluate genetic diversity and relatedness among the 28 kenaf genotypes using genetic similarity (GS). GS ranged from 0.36 to 0.97. The lowest GS (0.36) was observed between ACC-1589 and H046, while the highest (0.97) was found for 3 varieties T15, Xinhong95, and Cuba72, which originated from Hunan in the Shangdong Province of China and from Cuba. Taking a GS score of 0.45 as the threshold, the 28 kenaf accessions could be classified into 2 discrete clusters (Fig 4). Dendrogram of 28 Kenaf varieties based on cluster analysis of 72 ploymorphic EST-SSR markers Cluster I was the major group with 19 varieties, while cluster II contained 9 wild species and near marginal species.

## Discussion

### Characterization of the kenaf transcriptome

Recent developments in sequencing technology such as next-generation sequencing have enabled novel gene discovery and the development of EST-SSR markers on a genome-wide scale without the need for the complete genome sequence. As next-generation sequencing has low cost, and high efficiency and accuracy, it has been widely used to sequence transcriptomes and has been successfully applied to *de novo* transcriptome sequencing and assembly in many plants, e.g., bamboo, rubber, kenaf and ramie[24–27]. To date, however, we have accumulated relatively little information on the kenaf genome, and this comparative deficit has become an obstacle to the development of strategies to improve fiber yield and quality. In the present study, use of next-generation sequencing and Illumina paired-end sequencing technology enabled the characterization of the kenaf transcriptome and the assembly of a total of 71,318 unigenes. Approximately 78.7% of the unigenes had homologs in the Nr protein database, and their functions could be annotated by searches of public databases. These functions were also classified by COG and GO and the metabolic pathways were ascertained using the KEGG database. The results obtained here provide valuable sequence information that will aid further exploration of the major genes for important agronomic traits in kenaf, and enable greater understanding of the molecular mechanisms of bast fiber formation and development.

### Development of 9324 EST-SSR markers for kenaf

The construction of high-density genetic linkage maps is of great importance to molecular-assisted selection, the identification of genetic relationships among germplasm resources, and map-based cloning. To date, the comparatively low number of markers has made construction of a high-density genetic linkage map impossible in kenaf. Chen et al. [28]used SRAP, ISSR, and RAPD markers to construct a preliminary genetic linkage map for kenaf. However, this map contained 26 linkage groups, whereas kenaf has a haploid chromosome number of only 18. This situation suggested that a larger number of more effective markers needed to be developed.

In our study, 12,886 EST-SSRs were identified from the kenaf EST dataset, and 15.3% of these sequences possessed SSRs and 9324 EST-SSR markers were developed. A few primers may have been mismatched and, in such cases, some EST-SSRs might have failed to amplify because large introns were present within the target amplicon, or because the primers were designed across splice sites. In order to evaluate the quality of the EST-SSR primers designed in this study, we analyzed 100 randomly selected primers and found that 72 primer pairs could successfully amplified their target amplicons (72% success rate); thus, approximately three-quarters of the 9324 EST-SSR primers might be expected to successfully amplify their targets using PCR. Sixty-one of the 72 primers identified polymorphisms with 2 to 4 alleles among the 28 kenaf varieties. The 72 EST-SSR markers also allowed the analysis of genetic relationships and diversity among the 28 kenaf varieties. Our analyses show that the EST-SSR markers described here are of good quality. Additionally, the analyses demonstrate that we have successfully achieved the main aim of this study which was the large scale development of SSR markers for kenaf. These markers will provide a valuable resource for future genetic linkage map, QTL analyses and molecular marker-assisted selection breeding.

### Comparison of EST-SSR and SNP markers in kenaf breeding

Currently, with the development of next-generation sequencing, it is feasible to develop a large number of single nucleotide polymorphism (SNPs) and EST-SSR markers. The increased availability of SNP markers and the development of rapid and highly automated genotyping technologies have stimulated the use of these markers in preparing high-density genetic maps for QTL identification, and molecular marker aided selection and breeding. Different mutational processes govern allelic variation at SNPs and SSR loci, with the mutation rates of SNPs being several orders of magnitude lower than those of SSRs. As consequence, SNPs are typically biallelic, whereas SSRs have high allelic richness and heterozygosity levels[29]. SSR markers therefore have higher information content than SNPs. Yu et al.[30] suggested that a 10-fold greater number of SNPs were required to achieve the same level of analysis as SSRs; Van Inghelandt et al.[31] proposed a range between 7 and 11-fold.EST-SSRs are derived from coding sequences and, therefore, associated with functional genes that may influence important agronomic characters. Variations in the lengths of the EST-SSRs located in the coding region of a functional region might affect gene expression and gene function. EST-SSRs located in the protein coding regions of agronomically important characteristics can be developed into functional markers for kenaf breeding. Even if EST-SSRs are located in the 5′- and 3′-untranslated regions of genes, they are nevertheless tightly linked to functional genes and can be used for selecting and pyramiding agriculturally valuable alleles in a kenaf marker-assisted breeding program. Each type of marker has its particular advantages and disadvantages. Therefore, the decision on which type of marker to use depends on the nature of the research that is being undertaken.

## Conclusion

To the best of our knowledge, this study is the first to develop large-scale SSR markers for kenaf. These data will lay a solid foundation for the construction of genetic linkage map and for marker-assisted breeding of kenaf. In addition, the characterization of the kenaf transcriptome and the substantial amount of transcripts obtained will facilitate investigations of fiber growth and development in kenaf and will also be of value to novel gene discovery and functional genomic studies.

## Supporting Information

S1 Fig Length distribution of contigs. (TIF)

S2 Fig Length distribution of unigenes. (TIF)

S3 Fig Length distribution of All-unigenes. (TIF)

S4 Fig The numbers of tandem repeats. (TIF)

S1 Table Details of the 9324 EST-SSR markers developed. (XLS)

S2 Table EST-SSR markers used for PCR amplification. (DOC)

S3 Table Accessions used for diversity analysis. (XLS)
