# Supplementary figures and images for: Characterization of the Kenaf (Hibiscus cannabinus) Global Transcriptome Using Illumina Paired-End Sequencing and Development of EST-SSR Markers
Source: PLoS One. 2016 Mar 9;11(3):e0150548. doi: 10.1371/journal.pone.0150548 (PMC4784950; doi:10.1371/journal.pone.0150548)

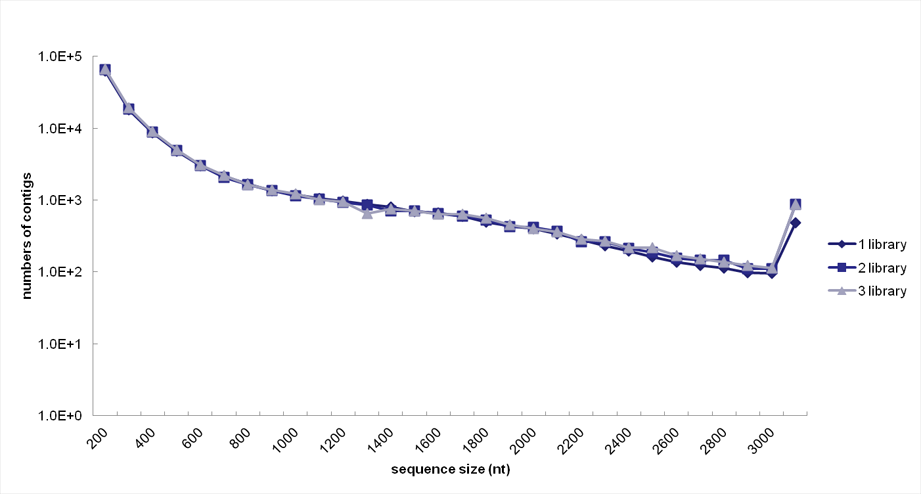

Supplement: S1 Fig — (TIF) [file pone.0150548.s001.tif]

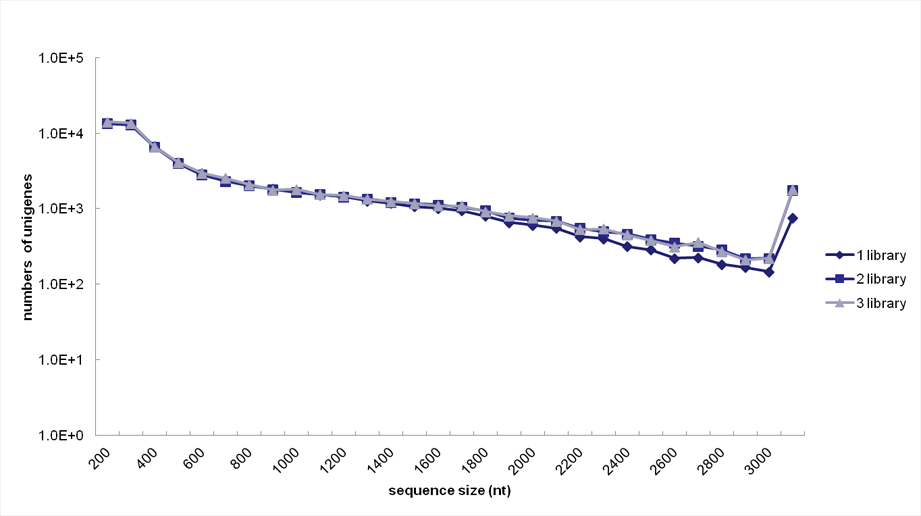

Supplement: S2 Fig — (TIF) [file pone.0150548.s002.tif]

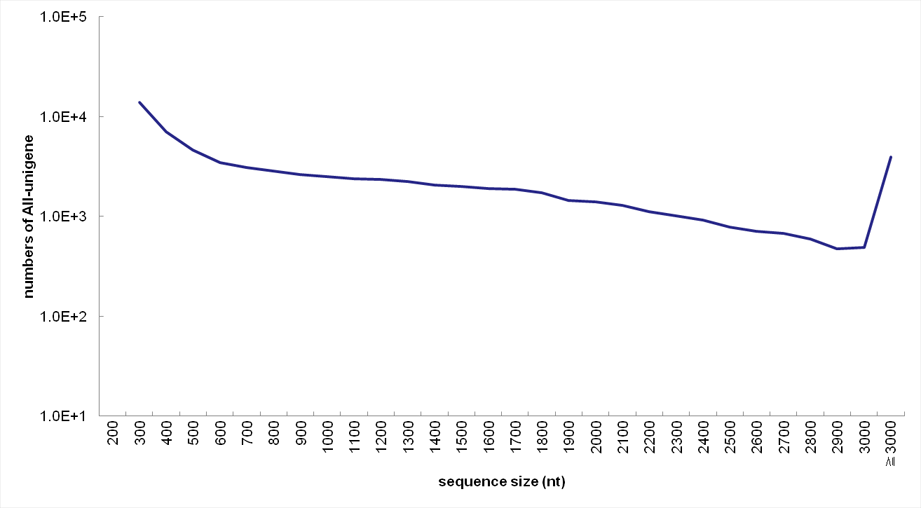

Supplement: S3 Fig — (TIF) [file pone.0150548.s003.tif]

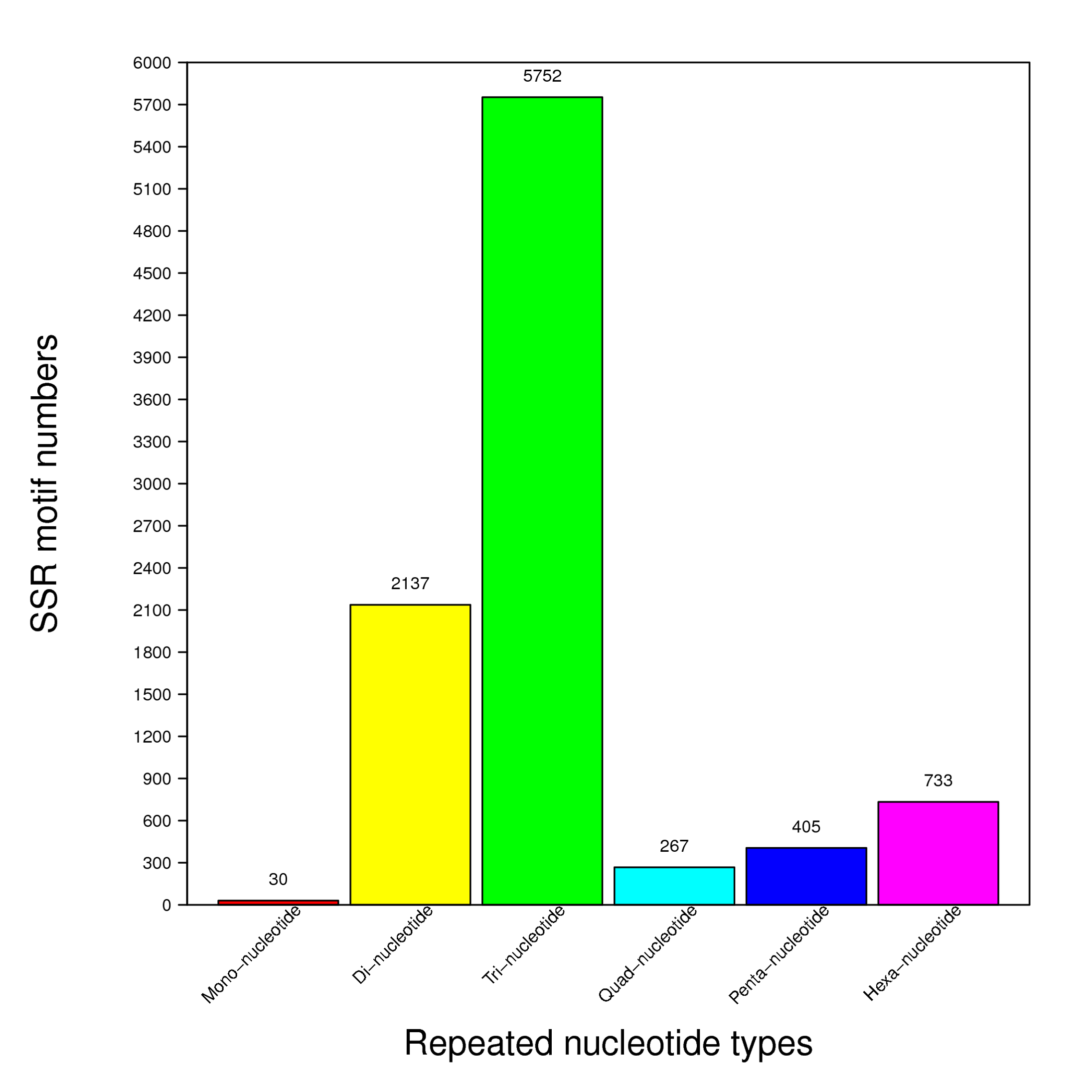

Supplement: S4 Fig — (TIF) [file pone.0150548.s004.tif]
