# Supplementary material for: Characterization of the Kenaf (Hibiscus cannabinus) Global Transcriptome Using Illumina Paired-End Sequencing and Development of EST-SSR Markers
Source: PLoS One. 2016 Mar 9;11(3):e0150548. doi: 10.1371/journal.pone.0150548 (PMC4784950; doi:10.1371/journal.pone.0150548)
Supplement: S2 Table — (DOC) [file pone.0150548.s006.doc]

Table S2. EST-SSR markers used for PCR amplification

| Marker | Primer sequence (5′–3′) |
| --- | --- |
| Kenaf-8 | F-primer: TCATCATCATTTTCCTCCTCATC  R-primer: ATCAACAATTTGGTCCTCCTGTT |
| Kenaf-18 | F-primer: TGAAGAAGAAGTGAAGGATGGTG  R-primer: GTGAAGAAGTTGGTCTGATCCAT |
| Kenaf-23 | F-primer: GCTGAGCTAAAACCAAGAGAAAA  R-primer: TTTTTGGACTGCTTCAGGATTAC |
| Kenaf-38 | F-primer: TGTAGCTGAAACTATCCCCAGAA  R-primer: CCTCATTTTTATTGCCAGTTGAA |
| Kenaf-46 | F-primer: CCTTTCCTCTTCTCCTTCTCCTT  R-primer: CGTGTTTCTGCATTGTTGTTTAC |
| Kenaf-62 | F-primer: CTGAACCCAAAACAAGAACAAGA  R-primer: CCATGTAATGGTGAGGGTACATC |
| Kenaf-72 | F-primer: GGTGATCCGTAAAACAAGTATGG  R-primer: GCATTTCTGAATTTATGCAAACC |
| Kenaf-82 | F-primer: ACTCTTCCTCATCATCTCCCAGT  R-primer: AGGGTGGCACCATTATATAGCTT |
| Kenaf-93 | F-primer: TCTCCAATACCCCTATCATCAGA  R-primer: CTGTCTCTGCCATGATTTTTCTT |
| Kenaf-98 | F-primer: CATCTTCTTCTTCCTCTTCACCA  R-primer: CTTTCTTCCATTCTTCCAAAACC |
| Kenaf-183 | F-primer: GAAGAGAACGAGATGGAAAATGA  R-primer: TCAGGATCAAGAGAGAAATCCAA |
| Kenaf-198 | F-primer: GAACAATTCAAGCACTCAATTCC  R-primer: TGGTCTACTCCAGAAAACAAAACA |
| Kenaf-442 | F-primer: GGTGCAACTTTCAGGTAGACAAA  R-primer: TTCTTCATCAAGACCCATACAGC |
| Kenaf-686 | F-primer: TTCGTTTCTCCTCCTTTCTCTTC  R-primer: GGAGGAGCATTAGATTTAGAGCC |
| Kenaf-786 | F-primer: TCCAACACTTCCAGCAAAAATAC  R-primer: ACTTTCCCTCCTCTTTTTCTCC |
| Kenaf-858 | F-primer: TAAGAGTAAGGGACAGGAGAGGG  R-primer: TAACAATCCTCCCAGCTCAAGT |
| Kenaf-946 | F-primer: ACCACCGATAACTTTGTCTGAAC  R-primer: ACTTCTCCAAGCTCACCCATCT |
| Kenaf-979 | F-primer: ACAAATCCTTTGCTTGCAGAAT  R-primer: CAATCCATGCCTTACCTTCATT |
| Kenaf-1070 | F-primer: CTACTACAGGTACGGATATGGCG  R-primer: TCAATTATATGCAACTCAGCAGC |
| Kenaf-1191 | F-primer: ATGTACTCTAATGGGATCTGGGC  R-primer: GTAATTTCCTTCCCACTCCCTTC |
| Kenaf-1366 | F-primer: AAAATCCTAATGGGACATATGGG  R-primer: TAAAACCACTTCCACCTTTCTGA |
| Kenaf-1456 | F-primer: ATAACAATCTGATGGGTTTCGAG  R-primer: CTGTGTATTTGCAGCATTTGTCT |
| Kenaf-1488 | F-primer: ATTTTGGGTTGTTAGCTGTTGAA  R-primer: TCAGCAGATTCTTGAACCTCTTC |
| Kenaf-1818 | F-primer: TTTCAAGGCATTGGATAACAGAC  R-primer: GATTAATCCATCAGCTTTTGCAG |
| Kenaf-1919 | F-primer: CATCAACTCCAGGTCTTTTGAAG  R-primer: TACTGAGCCAACCTCAGATAAGC |
| Kenaf-1988 | F-primer: TATTCTAGTCGTGGTATGGGTGG  R-primer: GAGTGCAACATGAAGAGGAATG |
| Kenaf-2116 | F-primer: AGATTACGTTATGGAGAAGGCGT  R-primer: TTTACCAGAAGCTGTACCTCCC |
| Kenaf-2376 | F-primer: TTCAACGTTCGTGTAAATGATCT  R-primer: CATTAAGGAATTGCTTGGACTTG |
| Kenaf-2456 | F-primer: AGCCTCATATATTCACATTTCCG  R-primer: CATTACTTTGAAGTTGACTCGGC |
| Kenaf-2555 | F-primer: TCTTTCTGTGGTTTCGTTTGATT  R-primer: ACTCTGTTTCCTTCCAACTTTCC |
| Kenaf-2666 | F-primer: AACCATTATTGGATTGTGGAGAG  R-primer: CAACGTGGTATCCTACACGTACA |
| Kenaf-2788 | F-primer: AAGTACGAAAGAATGTGACCTCG  R-primer: TACAACATTGAAGGGCAGTGTTT |
| Kenaf-2888 | F-primer: CCTTACATCCGAATCCACTTGT  R-primer: TTGTCTGAGAAGAGAGGAAGGTG |
| Kenaf-2999 | F-primer: GGAGACAAGTTTGACGACAGAAG  R-primer: AAACTCCCCTTCTCCTTTACTCC |
| Kenaf-3026 | F-primer: GTGTCTTAGGCAGCGTATCCA  R-primer: GAGAGACACGTGCTTTAAAATGG |
| Kenaf-3188 | F-primer: AAGGAAGCTTGAAAGAAGCATTT  R-primer: TTTGTTTAAAGATACCCGCAAAA |
| Kenaf-3226 | F-primer: AAATTGTAGGTGGCTGGGATTAT  R-primer: ACTTGGAAGAAGGAGGTGAAGAG |
| Kenaf-3299 | F-primer: GGGAACATTGTCTCTCTAGGGTT  R-primer: AACTCGAATTCACGCTTCAGAC |
| Kenaf-3333 | F-primer: TGGGAGGTCTTATTACCCAGTCT  R-primer: GTTGCACAGGTATATTGTAGGGC |
| Kenaf-3466 | F-primer: TCAAAACCTCGCTGAAAGAGTTA  R-primer: AAACGTTATCAGAATCAGACCCA |
| Kenaf-3666 | F-primer: AGCACTCATGAAAAACCACTCTC  R-primer: AAGCCGTGTCTTTCTCCTTAATC |
| Kenaf-3888 | F-primer: AGCTTTAAGCAAGTGGTCCTATG  R-primer: AACACAGGGGAAAACTGTACAAA |
| Kenaf-3988 | F-primer: TGGTGATCCTGCATAATCTTACA  R-primer: TGGATGTCTTCTCTTCAGACACA |
| Kenaf-4071 | F-primer: CATCTTCCTCCAGTAATGATTCG  R-primer: ATAACTCATCTGTAAGCCACGGA |
| Kenaf-4108 | F-primer: TGGCGTTATTGTTGGTTTTTAGT  R-primer: CACCATATGGATCAGCTTTAGGA |
| Kenaf-4208 | F-primer: TAAGCAAGAAAGAAGCAAATGGA  R-primer: GAATTCGGTAAATGCGAGTTATG |
| Kenaf-4369 | F-primer: GAGAAGGAACAGACATTTGCAGT  R-primer: GAGACAGTGGAGCTCTGAAACAT |
| Kenaf-4444 | F-primer: TTGCTCTCGGCTCTACTTCTATG  R-primer: GACAACGTAGGGGTTAGTGAGTG |
| Kenaf-4556 | F-primer: AGACTTTTATTGCATGCGGTACT  R-primer: ATCATCCTTAAGCTTTCGTAGCA |
| Kenaf-4586 | F-primer: TTACCTTCATATAGGCGGAAACA  R-primer: TCGAGACCCTAGCCTTAACTTTC |
| Kenaf-4666 | F-primer: ATCAGAAGTTGAAGCAGAAGTCG  R-primer: GGTTGAAGCAGACTCTGACAGTT |
| Kenaf-4776 | F-primer: TTATTGATCTCGGAGGCTAAGTG  R-primer: GCTGCTTTTCACCCCATTATC |
| Kenaf-4888 | F-primer: TGACCACTGCTATACTTCTGCTG  R-primer: TGATCTGAACCGAGTGATTTTCT |
| Kenaf-4936 | F-primer: GGAGTTTTGAATGTGAACGAAAG  R-primer: TCAACTTTTTCAACCATAAATTGAAC |
| Kenaf-4986 | F-primer: AGGTCTAAATATGGAGGGACACC  R-primer: CCAGCTCTGCTCTGATAGCTAAA |
| Kenaf-5016 | F-primer: ACTCACCGTCCTTACTTCTTTCC  R-primer: AGTTTCATACTTTGGAGATGCCA |
| Kenaf-5116 | F-primer: CGACTTTAAGCAAAATCCACAAT  R-primer: AAGGAAATGAAGCTCGAGAAAAT |
| Kenaf-5136 | F-primer: GTCTTCTTCACCACCATCTTCC  R-primer: GAGTCGACACTTCTCCTGTGTTT |
| Kenaf-5146 | F-primer: CCTCTCCCAGTCCCTAATTACTC  R-primer: GAAATCGGCAAAGGAGGATT |
| Kenaf-5156 | F-primer: TTTCGTTGAGATCTCTCGAGTTG  R-primer: GGAGCAATAGAACTGTTTCCTCA |
| Kenaf-5216 | F-primer: GAGCTGATCTGATGTGATGAAAA  R-primer: TTATTTCAGAGAATGAAACCAAA |
| Kenaf-5258 | F-primer: TTCAGCTTAGAAGACTTGGCTTG  R-primer: CGAGTAACGGTTTGAGTTTGC |
| Kenaf-5320 | F-primer: CACAAAAATACACATTTGGGTCA  R-primer: TCACAAGGAATTTTGTGTCCACT |
| Kenaf-5348 | F-primer: TGCTTTCAACACTTAAAAGATGG  R-primer: CATCCGATTCAGATTAACTCCAG |
| Kenaf-5438 | F-primer: GTTTAACTCCATGGCAGAACG  R-primer: TTGAACTGGGTGTTTTTCAGAAT |
| Kenaf-5476 | F-primer: TTCTCATACCCGTAGACAGTCCT  R-primer: AGATGTCAGAGCTGAGATTGGTC |
| Kenaf-5548 | F-primer: TTCCCAAAGAAAACCAACTTCTT  R-primer: GAAATCTGAAATGGAGAATGGTG |
| Kenaf-5598 | F-primer: TTGACAATCAGAGTGTTGCAGTC  R-primer: AAAATTCTCTCTTGCTTTGTCCC |
| Kenaf-5636 | F-primer: TTAGTCACAAAACCCAACAGCTT  R-primer: TTTTTCTTCTTCGACTTCACTGC |
| Kenaf-5676 | F-primer: CTGCTATCATGCCAACTAGCTTT  R-primer: GCCTGTTAAAATGGATCAACAAG |
| Kenaf-5771 | F-primer: GCTTCTCGCCATTGTAGTAGTTG  R-primer: GAATCCATGTTCACAGAGAAAGG |
| Kenaf-5856 | F-primer: GAATCAAATTGTGAGGTCAGGAG  R-primer: GATAAAGATGATCGTGGTGGTCA |
| Kenaf-5890 | F-primer: TCAGATTCCTCTTTCTCCTCAAC  R-primer: TAACTGAGCTTATTGCATCTGGG |
| Kenaf-5970 | F-primer: GCCTAGAGAAGTGAGGCTTTTGT  R-primer: ACTTGAAAACACCCAACAGACC |
| Kenaf-6000 | F-primer: CTGAAGGGAAGTAGGGAGATGAT  R-primer: TAAAGTCAAACTGCTCCATCCAT |
| Kenaf-6011 | F-primer: TTCATCATGTGTTGTAAGGCAAC  R-primer: TGGAAAGATTGATGAGAGAAGGA |
| Kenaf-6108 | F-primer: CAGAAATAGCGACGAGAAAATCT  R-primer: CTGTTGCTGCTGTTGTTATCTTG |
| Kenaf-6140 | F-primer: ATATAAGAAAAGGAGGTGGAGGC  R-primer: AAATGGAAGGAAGAAATATGGGA |
| Kenaf-6326 | F-primer: AGATCCTGTTTTTGTCATCCAGA  R-primer: AACACCATGGAAATGATGTTTTT |
| Kenaf-6416 | F-primer: ATCTGAACCGAGTGATTTTCTCA  R-primer: TGACCACTGCTATACTTCTGCTG |
| Kenaf-6528 | F-primer: TAACCTTTGCACAGGAAATGAGT  R-primer: ACAAAATACAAGCTGTCTGCCTC |
| Kenaf-6666 | F-primer: ACTTCTCTAACTCAATCGACCCC  R-primer: TACCAACTCGTCCTCTGTCATTT |
| Kenaf-6770 | F-primer: AATAGGATCATGGTCTTCTGCAA  R-primer: TACTCATCAGAATCCCCAGAAAC |
| Kenaf-6888 | F-primer: CCTCAGTTGCCATATCCATAATC  R-primer: GATCTTCCAAAGGAGGAGAAGAA |
| Kenaf-6968 | F-primer: CTTTTGTGTTTTGATGCTTTGAA  R-primer: AAACGAATAGCTGCAGAATTGAA |
| Kenaf-7070 | F-primer: TATTCCCTGCAAACAAAAAGCTA  R-primer: GTATACCCACAGGCTTTACCCTC |
| Kenaf-7388 | F-primer: CAAGATGGATACACTATGGGGTC  R-primer: TTTGACATATGAAGATGCCAGTG |
| Kenaf-7427 | F-primer: CCTCCACCTATGTTTTGTTTTTG  R-primer: TTTCTTCTTTACCGTACACCCCT |
| Kenaf-7555 | F-primer: ATGCTTCTTTTCTGTTTTTGCAG  R-primer: AAACCTAATCCCTCCTAAACCCT |
| Kenaf-7666 | F-primer: GCAAGAGACATGAGACCAAAGAA  R-primer: ACATTTATACTCGCGAACCACAT |
| Kenaf-7777 | F-primer: AACAGATAGATAAACGAACGGCA  R-primer: GAGAAAACGCCGTTAAGTTACAG |
| Kenaf-7858 | F-primer: AAGGTGAACTGATCGAACTGAAC  R-primer: GCATGAAAAGTACAAGGACAAGG |
| Kenaf-7896 | F-primer: GTTTTGTCTAAGTTGCTTGTGGC  R-primer: TAAAATTTCTCTTTGAACCTCGG |
| Kenaf-7928 | F-primer: GGAAGAAGCTTCCGAAGGAAT  R-primer: TCTTGTAGGTCTCAACGCTCTTC |
| Kenaf-7999 | F-primer: AAAGGGAAATTCAATCAACTGGT  R-primer: ACCGGTAAACGAGAACAATTACA |
| Kenaf-8036 | F-primer: CACAGAAACTAGCTGCGAGAAAT  R-primer: TTCCTGTGGGTTATAGGAAAACA |
| Kenaf-8088 | F-primer: TTGGGTAGTGGTACTCATTGCTT  R-primer: CCTGATTATCTGCTTCAACAACC |
| Kenaf-8466 | F-primer: CTGTTTGATGACCGTAGGAAGAG  R-primer: CATTATCAGTACGAACCACGACA |
| Kenaf-8666 | F-primer: AAAGTGGATTTATGAGCGGTTTT  R-primer: TTGGTGTCAAAATTACCCTTCTC |
| Kenaf-8888 | F-primer: AAGATGGATTAAGCAACGAACAG  R-primer: TCACCAGGATTTGCTTTTATCAT |
